# Conducting epidemiological studies on snakebite in nomadic populations: A methodological paper

**DOI:** 10.1371/journal.pntd.0011792

**Published:** 2023-12-28

**Authors:** George O. Oluoch, Denis Otundo, Seth Nyawacha, Derick Ongeri, Monica Smith, Vivianne Meta, Anna Trelfa, Sayem Ahmed, Robert A. Harrison, David G. Lalloo, Ymkje Stienstra, Frank-Leonel Tianyi

**Affiliations:** 1 Kenya Snakebite Research and Intervention Centre, Kenya Institute of Primate Research, Ministry of Health, Karen, Nairobi, Kenya; 2 Centre for Snakebite Research and Interventions, Liverpool School of Tropical Medicine, Pembroke Place, Liverpool, United Kingdom; 3 Department of Tropical Disease Biology, Liverpool School of Tropical Medicine, Pembroke Place, Pembroke Place, Liverpool, United Kingdom; 4 LocateIT Limited, Galana Road, Nairobi, Kenya; 5 Health Economics and Health Technology Assessment, School of Health and Wellbeing, University of Glasgow, Glasgow, United Kingdom; 6 University of Groningen, University Medical Centre Groningen, Department of Internal Medicine/Infectious Diseases, Groningen, The Netherlands; Fundação de Medicina Tropical Doutor Heitor Vieira Dourado, BRAZIL

## Abstract

**Introduction:**

Research on snakebite has mostly been conducted on settled populations and current risk factors and potential interventions are therefore most suited for these populations. There is limited epidemiological data on mobile and nomadic populations, who may have a higher risk of snakebite.

**Methods and results:**

We conducted a scoping review to gather evidence on survey methods used in nomadic populations and compared them with contemporary survey methods used for snakebite research. Only 16 (10.5%) of 154 articles reportedly conducted on pastoralist nomadic populations actually involved mobile pastoralists. All articles describing snakebite surveys (n = 18) used multistage cluster designs on population census sampling frames, which would not be appropriate for nomadic populations. We used geospatial techniques and open-source high-resolution satellite images to create a digital sampling frame of 50,707 households and used a multistage sampling strategy to survey nomadic and semi-nomadic populations in Samburu County, Kenya. From a sample of 900 geo-located households, we correctly identified and collected data from 573 (65.4%) households, of which 409 were in their original locations and 164 had moved within 5km of their original locations. We randomly sampled 302 (34.6%) households to replace completely abandoned and untraceable households.

**Conclusion:**

Highly mobile populations require specific considerations in selecting or creating sampling frames and sampling units for epidemiological research. Snakebite risk has a strong spatial component and using census-based sampling frames would be inappropriate in nomadic populations. We propose using open-source satellite imaging and geographic information systems to improve the conduct of epidemiological research in these populations.

## Author summary

Nomadic populations are at increased risk of snakebites, but interventions will not be developed or improved without reliable data on this risk. Highly mobile populations require specific considerations in selecting or creating sampling frames and sampling units for epidemiological research, and using census-based sampling frames would be inappropriate. We conducted scoping reviews to gather evidence on survey methods used in nomadic populations and compared them with contemporary survey methods used for snakebite research, which we used to inform our methodology. We describe the creation of a sampling frame of 50,707 households, the selection and geo-tagging of a sample of 900 households, and the identification and enrolment of participants from 875 households between August 2019 and March 2020. We propose using open-source satellite imaging and geographic information systems to improve the conduct of epidemiological research in these populations.

## Introduction

Snakebite envenoming is a major neglected tropical disease that can cause acute life-threatening effects and long term physical and psychological disability [1,2]. An estimated 5 million people are bitten by snakes each year, the majority occurring in tropical parts of the world [3]. Both human-related and snake-related factors influence the chance of an interaction between a human and a snake and hence the occurrence of snakebites, their pathological severity, and long-term implications. Whilst the relative abundance of both human and snake populations are important predictors of a snakebite event, the presence of humans may also cause a decrease in the abundance of snake populations, often resulting in an inverse relationship between human population density and snakebite [4,5].

Most predictors of snakebite have been identified and described using surveys, epidemiological and modelling studies, which often use sampling methods based on a fixed residence. They assume that individuals reside within an administrative unit, a geographic space, a house, or a physical structure, and that each household represents a fixed domestic unit [4,6].

These assumptions do not hold for mobile populations such as nomadic pastoralists who inhabit the arid and semi-arid parts of West and Eastern Africa. Their nomadic lifestyles involve regular migration, seeking grazing land and water for their livestock in lands dominated by patchy seasonal rainfall [7]. Such lifestyles increase the risk of settling in snake-abundant locations and thus increase the risk of snakebites. Furthermore, nomadic pastoralists live in rural areas, and they engage in cattle herding, both of which are known to increase the risk of snakebite, making nomadic populations a particular interest group for snakebite research [4]. Their mobility limits their access to healthcare workers and engagement in research studies, and has resulted in nomadic populations being historically under-represented in population censuses, surveys, and epidemiological research activities, with some authors labelling them as being “statistically invisible” [8]. This lack of epidemiological data suggests there may be a limited appreciation of the burden of snakebite in nomadic populations, thereby delaying the design of snakebite prevention interventions adapted to a nomadic way of life.

With less than a decade remaining to achieve the WHO’s ambitious goal of halving the morbidity and mortality of snakebite envenoming [9], it is crucial to explore novel epidemiological methods and tools to better describe the burden of snakebite in high-risk groups like nomadic pastoral populations.

This paper describes our experience conducting an epidemiological survey on nomadic and semi-nomadic populations in Samburu County, Kenya. We reviewed studies conducted on nomadic populations, and recent publications in the snakebite field to understand contemporary study designs, and to highlight the differences with the methods we used. We share lessons learnt from this experience, and we discuss the potential benefits of this novel approach for conducting epidemiological research in highly mobile populations. The study was undertaken as part of the multidisciplinary research objectives of the UK National Institutes of Health Research (NIHR) funded African Snakebite Research Group [10,11] and was the methodology used in our publication estimating snakebite prevalence and mortality in Samburu County [12].

## Methods

### Ethics statement

Ethical approval for the survey component of this study was obtained from the Kenyatta National Hospital–University of Nairobi Ethics Research Council, Nairobi, Kenya (P195/04/2017) and the Liverpool School of Tropical Medicine Research Ethics Committee (Research Protocol 18–058). No original data on individuals were included in this methodological study. The findings of snakebite prevalence and mortality in Samburu County, including the consent procedure followed, are described elsewhere [12].

### Study population and setting

Samburu County is located in the Northern part of Kenya, 300 km from the capital Nairobi, and lies within the Great Rift Valley. It has a surface area of 21,022 square km, with an estimated 319,708 inhabitants in 2017. The county is divided into three sub-counties, 7 divisions, 14 locations and 106 sub-locations, with 17.1% of the population living in urban settings [13]. Samburu is home to various pastoralist tribes, including the Samburu, Turkana, Rendile, and Borana tribes. Pastoralism is a major source of income, with 8 out of 10 households keeping livestock. Rainfall patterns are highly variable, ranging between 250 to 700mm in the plains and 750mm– 1250mm in the highlands, and temperatures range between 24°C in July and 33°C in December meaning that Samburu county has an arid or semi-arid environment [14].

The pastoralist communities comprise a heterogeneous group of different ethnicities and other differences, including languages, social organisations, degrees of mobility, sedentariness, livestock ownership, and economic diversification. Pastoral activity lies on a continuum from sedentary pastoralists who engage in agriculture and other economic ventures, to mobile pastoralists or nomads who practice transhumance with all their livestock [8, 14]. The basic social unit is the nuclear family, which could be monogamous or polygamous. Some families split up at certain times of the year, and the *Imurrani* (unmarried young adult men) undertake long distance migration with the livestock, while the rest of the family remains in a more-or-less fixed residence from which they look after sheep and goats. The homes or settlements are referred to as *nkang* or *manyatta*, and they consist of a single circular hut, or multiple huts arranged in a circular manner with a space in the middle, surrounded by an acacia thorn fence. The houses are inexpensively constructed with muds and sticks that can readily be abandoned (*midjoni* [abandoned homestead where there are still some structures standing]) in case of conflict or to look for grazing land for the remaining livestock. Some families could be split kilometres away at the same time, in different counties or countries, despite constituting the same nuclear family [15, 16]. This can have consequences for defining sampling frames and sampling units for population health research.

### Scoping review of epidemiological research in nomadic populations and on snakebite victims

We conducted a search of the PubMed database to gather evidence on survey methods used in nomadic populations and compare them with contemporary survey methods used for snakebite research. We followed the protocol for conducting scoping reviews as described in the Manual for Evidence Synthesis [17].

Our search words for the nomadic populations included “nomad*”, “pastoralist” or “transients and migrants”, with alternating combinations of Medical Subject Headings (MeSH) and free-text words, and we included surveys or cross-sectional studies conducted in African countries. The search returned 792 articles, of which 154 were population-based surveys involving pastoralist nomadic populations. Our review showed that most studies labelled as being conducted in nomadic pastoralist populations, were actually conducted on settled pastoralist populations who have transitioned in favour of sedentary agricultural activities, have fixed and permanent residences, and for whom contemporary study designs were appropriate. Only 16 (10.5%) [18–33] articles involved mobile pastoralists who engage in long distance herding and seasonal migration in search of grazing land, and we have summarised the main epidemiological methods used to study these mobile pastoralist populations in Table 1.

Our search words for surveys on snakebite included “snakebite*” or “snake envenoming*” or “snake bites”, with alternating combinations of MeSH and free-text words, without any geographic or language restrictions, and we considered surveys or cross-sectional studies published between 2012 and 2022. The search returned 184 articles, of which 18 [34–51] were community-based cross-sectional surveys and were included in our review. Detailed search strategies and the results of both reviews are provided as supplementary material (S1 and S2 Tables, and S1 and S2 Figs). All 18 studies used a multistage cluster sampling design with variations in the sample size calculations, number of sampling stages, sampling frames, and the selection of the primary sampling units (PSU) and the final sampling units (FSU). The most common approach was to select clusters (PSU) from a population census sampling frame using the probability proportional to size (PPS) approach, and to randomly select households from each cluster in the final sampling stage (FSU) [4, 6, 35, 37, 39].

**Table 1:** Epidemiological methods used to study nomadic populations and examples of a study using the approach.

Method	First author, Year, Study location	Brief description of method
Camp approach	Jean-Richard, 2015 [52] Chad	Nomads in an area are grouped into camps, based on their proximity to one another, and a list of these camps is created with an accurate description of their location. This list constitutes the sampling frame from which camps will be selected for inclusion in research activities, and the primary sampling unit is the camp. It uses a *de jure* approach where people are enumerated based on their usual residence
Enumeration area	Hazel, 2015 [22] Namibia	Specific geographic locations where nomadic populations usually settle are enumerated, specifying their geopolitical boundaries, and used as sampling units. A list of these locations comprises the sampling frame. The primary sampling unit is a geographic location, and nomadic populations settled in these areas are identified and interviewed. It uses a *de facto* approach, where people are enumerated based on where they are on the day of the census.
GIS methods	Wild, 2019 [7] Ethiopia	This involves using geospatial techniques to identify settlements. The settlements are grouped using geospatial software to form a sampling frame of clusters and the clusters are the primary sampling units. Clusters can be selected using PPS. A list of all households in each cluster is drawn, and some are randomly selected as secondary sampling units. Nomadic populations living in the selected households are identified and interviewed
Group assembly	Macpherson 1989 [25] Kenya, Sudan, Ethiopia, Tanzania	Study teams, with assistance of administrative authorities, assemble all nomads in their administrative units to a specific point where they are interviewed. It is an extension of the census-approach and all nomads in a specific area at a specific time make up the sampling units.
Social structure approach	Jacques Brenner, 1971 [53] Mauritania	This uses existing social structures and hierarchies to organise nomadic populations into different sampling units. The approach relies on the authority of the tribal leaders to recruit members of their communities for research purposes. Social subdivisions such as tribes, subtribes or clans can represent different sampling units depending on the aim of the research. Nomadic populations belonging to each social structure are identified and interviewed
Water point approach	Bawa, 2018 [33] Nigeria	Waterpoints at which nomadic populations and their livestock seek water are identified and constitute the sampling units. Nomads who use the sampling points are invited to participate in interviews.

## Epidemiological methods used to survey nomadic and semi-nomadic populations in Samburu County, Kenya

### Sampling frame creation

We created a spatial sampling frame by directly identifying and creating data points for all features that looked like a household within the 2018 level 1 global administrative unit boundary for Samburu County, using high-resolution satellite images. Images were downloaded from the Copernicus Sentinel-2 Earth observation mission of the European Space Agency [54]. The mission consists of two polar-orbiting satellites which monitor variability in land conditions by continuously capturing high resolution optical imagery (10m spatial resolution) over land and coastal waters with 5 to 10 day revisits [54]. The open-source data is provided as 13 band multispectral data in the visible, near infrared and short-wave infrared portions of the visual spectrum. We combined the images from the visible red, green, and blue bands to generate a true colour composite image of all visible structures in Samburu County, with vegetation appearing green, and bare surfaces appearing light brown to grey. Images from mid-August 2019 to mid-September 2019 were merged (mosaiced) to produce a single (medium) image which was used to create our sampling frame.

**Fig 1:**
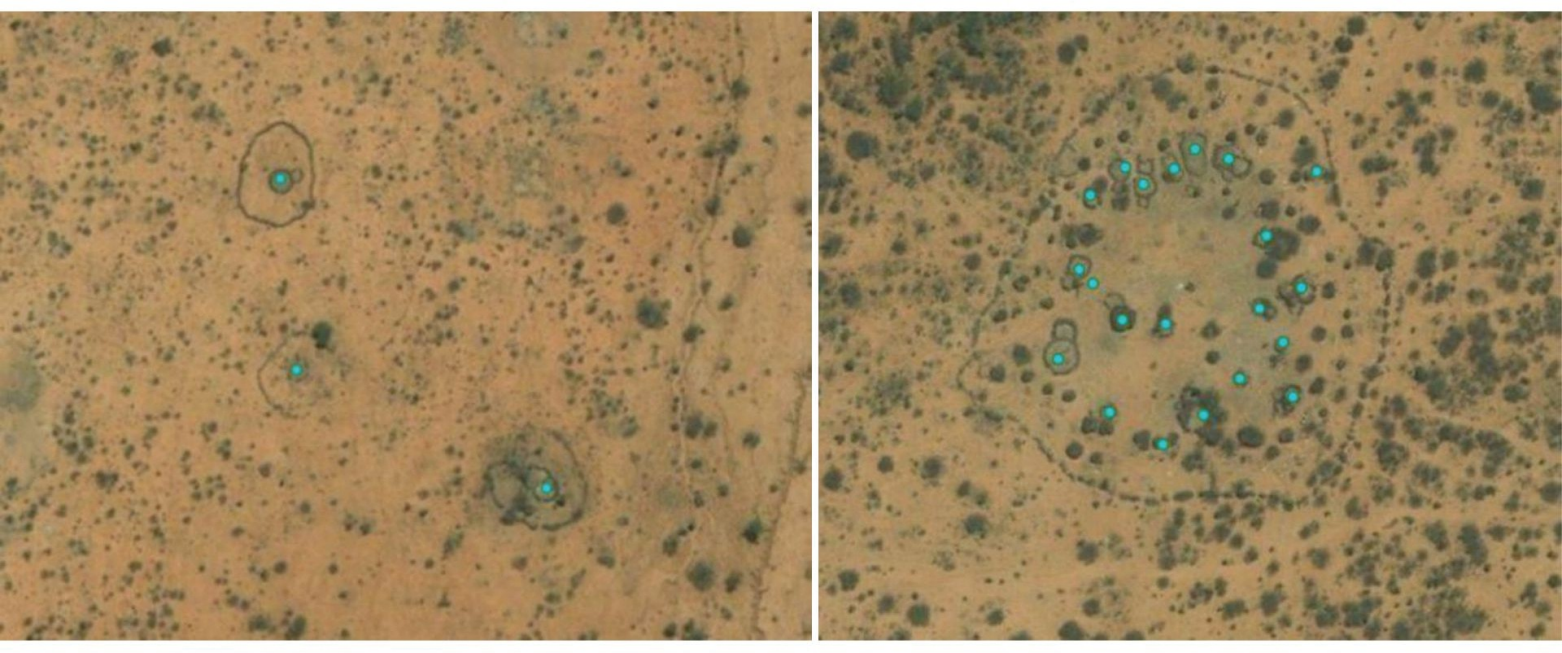
**Spatial images of homesteads in Samburu county, Kenya.** A. Spatial image of three single homesteads with surrounding thorn fences. B. Spatial image of a nkang or manyatta

In Samburu, families live in bomas or homesteads (physical structures in which people live), which are low circular houses made with sticks and mud. They have a characteristic spatial image and Fig 1A shows a stand-alone homestead which is usually surrounded by an acacia thorn fence to keep livestock in and wild animals out. Several homesteads could be grouped together into *nkangs* or *manyattas*, with a single thorn fence around the grouped homesteads (Fig 1B). In semi-nomadic areas, the homesteads had a different spatial appearance–rectangular iron sheets. We identified 50,707 homesteads from the whole of Samburu, and these were all manually digitised and converted into datapoints using ArcGIS.

We used a modified grid-based GIS sampling method to select our primary sampling units [55]. Our sampling unit in this study was the grid, with each grid serving as a proxy for the smallest administrative unit. In Kenya the smallest administrative unit, is a village. We estimated the size of each village to be 25 square kilometres and divided the entire Samburu County into 5 by 5-kilometre grids, generating 956 grids. We chose a 5x5km grid size because it allows us to capture more details, especially about spatial patterns of homesteads/houses in each grid, thereby enabling a more accurate estimate of the burden and distribution of snakebite in our study area.

### Sampling strategy

The cluster grids and the mapped homesteads were merged to create a gridded household dataset which was exported as an excel sheet alongside the global positioning system (GPS) coordinates of the homesteads. We then calculated the total population per grid using an average of eight members per household as outlined in the Samburu County Integrated Development Plan [14]. A PPS approach was used to select grids for inclusion in the study. The selected grids were noted, 25 homesteads were randomly selected from each grid, using the random generator tool in ArcGIS known as ‘Random Subset’ that applies the Mersenne Twister random generator method [56]. We used the amount of vegetation within the homesteads to differentiate inhabited from uninhabited households. We assumed that inhabited homesteads kept grazing livestock, and these would consume the vegetation within the thorn picket, hence inhabited households had little vegetation while uninhabited homesteads had overgrown vegetations. We recorded the geospatial coordinates of the selected grids and inhabited homesteads within the grids.

### Navigation to selected households

The GPS coordinates of the selected grids and the selected homesteads were loaded into a GIS based mobile application provided by the Environmental Systems Research Institute (ESRI) East Africa called *Collector* [57]. Data collectors downloaded the Collector App on their data collection tablets and used this to navigate to the selected points. The app has both online and offline functionality and allowed navigation in areas with poor network coverage.

### Sample size

#### We considered three methods for calculating our sample size

- (i) The first approach involves a conventional sample size calculation using prevalence (incidence) estimates from previous studies, a pre-determined precision and 95% confidence intervals, and adjustments for the clustered design and for non-response. These are standardised multistage cluster survey methods which are used by stakeholders such as United Nations Children’s Fund (UNICEF) and the United States Agency for International Development (USAID) to conduct large scale population surveys at a national level [58, 59]. This was the most common method of sample size calculation in recent snakebite surveys [6, 35, 37]. This method is robust and replicable, and allows for comparability with other studies, however because it depends on incidence estimates, rare diseases like snakebite (incidence < 1%) will have very large sample sizes, requiring significant funding and resources.
- (ii) The second approach involves using a fixed proportion of the study population and adjusting the sampling strategy accordingly. It is less robust and offers less comparability compared to the previous method. Nonetheless, it allows for a practical and straight-forward readjustment of the required sample size to align with existing research funds and resources [39].
- (iii) The third approach involves conducting a survey of the entire population and collecting data on the disease of interest, often via a pre-established disease surveillance system. This approach provides the closest approximation to the true population estimate but is limited by prohibitive setting up and running costs. An example is the Kilifi Health and Demographic Surveillance System that was established in Kilifi, Kenya in 2000 and which routinely collects data on births, pregnancies, population movements, and deaths. By linking this data to hospital records and adding epidemiological questions to the routine surveys, the Kilifi Health and Demographic Surveillance System facilitates the conduct of hospital- and community-level surveys on acute and chronic diseases, with important findings on childhood infections and the prevalence of epilepsy in Kilifi [60].

### Inclusivity in global research

Additional information regarding the ethical, cultural, and scientific considerations specific to inclusivity in global research are included in the Supporting Information (S1 Checklist).

## Results

Data collection was carried out between December 2019 and March 2020, three months after completing the geospatial cluster and household mapping. The study was conducted in Samburu County, Kenya, and we recruited participants from all three sub-counties in Samburu.

### Sample size

We calculated a sample size of 900 households, which represented 2% of the households listed in the 2009 census in Samburu. To accommodate funding and logistical constraints, assuming that our data collectors could visit 5 households in a day, we settled on selecting 36 clusters and 25 households per cluster. We adopted the definition of a household from the Kenyan Bureau of National Statistics which considers everyone who spent the previous night in the household as a member of the household [61]. We will use the word household to reflect both the social organisation and the physical structure in which participants resided.

### Grid and household identification

**Fig 2:**
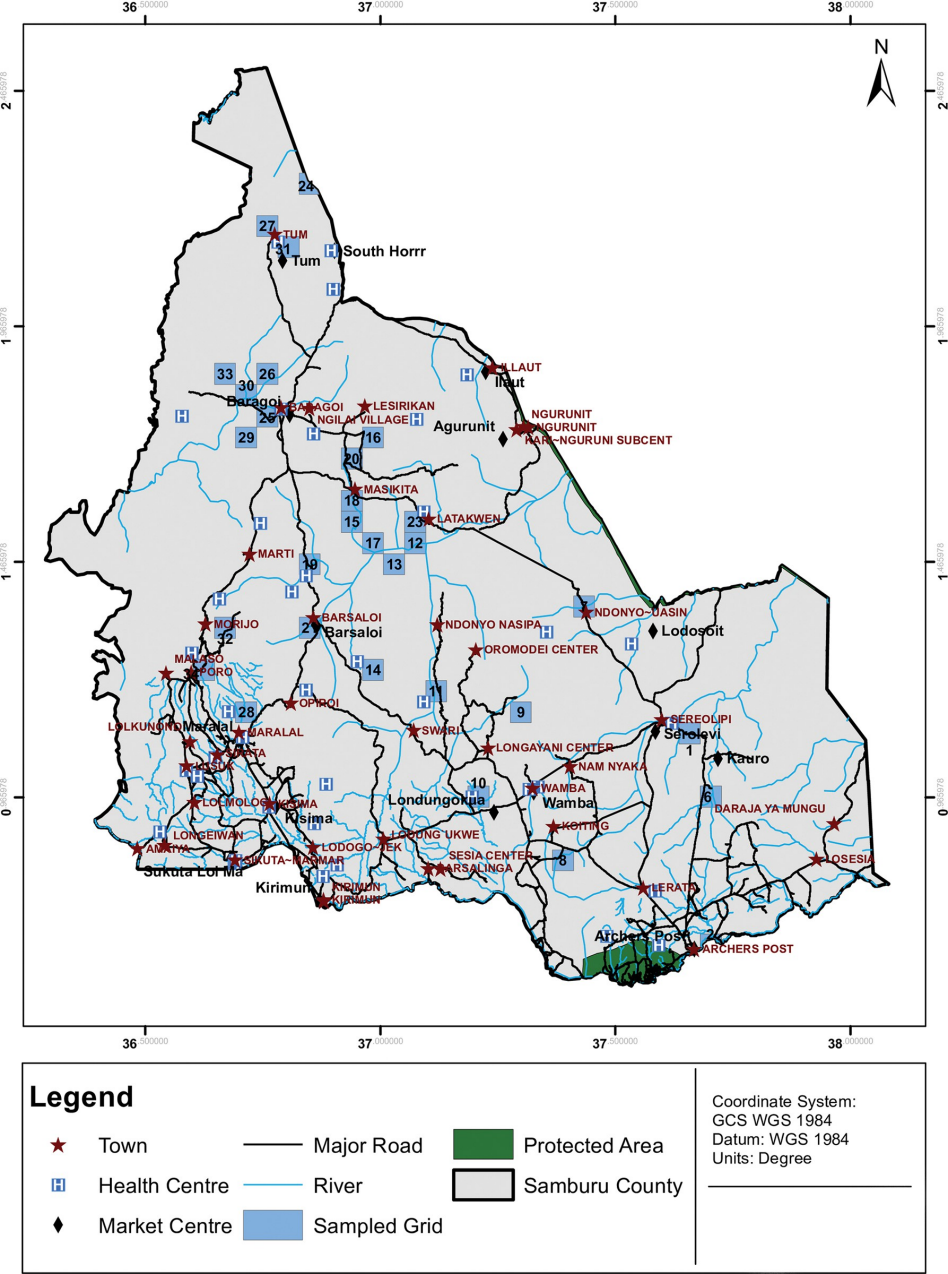
Map of the Samburu County grids and selected grids following probability proportional to size sampling (*Created using ArcGIS*, *base map from https://gadm.org/)*.

A sampling interval (SI) of 11268 was obtained by dividing the total estimated population of Samburu by 36. A random number between 1 and the sampling interval (SI) was generated to obtain our Random start (RS). The random number obtained from https://www.random.org/) was 3374. The random start series: (RS; RS + SI; RS + 2SI; RS + 3SI; RS + 4SI;…RS + 36SI) was computed with the RS and SI being constant. Thirty-six grids where the cumulative population size contained the numbers generated in the series were selected for sampling. Twenty-five households were randomly selected from each grid, and each household had the same probability of being selected. Fig 2 show the grids that were selected using the PPS approach.

There was an even distribution of selected grids across all sub-counties in Samburu. All grids were identified during the data collection period and data collectors successfully navigated to the 36 selected grids. Navigation to the household locations were successful as well. However, there were significant population movements in some areas, and in extreme cases, all of the inhabitants of some grids had moved to another location. In some instances, we followed some households across grid boundaries, especially when they had moved within a radius of 5km. We accounted for this by assuming that their originally mapped location was their true place of residence and relocating these inhabitants to their original grids. In instances where the inhabitants had moved beyond a radius of 10 km, we replaced the original household with a new household in the same grid. For the completely abandoned grids, we oversampled the same number of households from nearby grids. Household identification and data collection occurred as planned in 18 (50%) grids. In 10 (27.7%) grids, geolocated households had moved from their original locations and were within a 5km radius (within or out of their original grid), hence these households were relocated to their original grids. In two (5.6%) grids, households had moved beyond 10km, and these households were replaced, while in six (16.7%) grids, there were no households at all, so nearby grids were oversampled accordingly.

We correctly identified 573 (65.4%) households, of which 409 (46.7%) were in their original locations and 164 (18.7%) had moved within 5km of their original locations. 302 (34.6%) households were oversampled to replace the completely abandoned grids and the grids where inhabitants had moved beyond 10km.

### Data collection

The household surveys were carried out by twelve trained local data collectors, grouped in teams of three, and one coordinator. They were assisted remotely and on-site by two GIS navigation experts. All data collectors and the coordinator travelled to one grid and completed data collection before moving to the next grid. Data collection tools were designed using KoboToolbox which is a free and open-source software used to design questionnaires, deploy surveys, and collect and review data. It can be linked to KoboCollect app which is freely available on google play and can be downloaded on laptops and mobile devices.

### Data outcomes

The epidemiologic burden of snakebite in Samburu County has been estimated from data collected using our approach, and a manuscript with the results has been published separately [12].

## Discussion

Correctly identifying the denominator is a key aspect of epidemiological studies. In community-based surveys, this depends on two main factors; the sampling frame for the primary sampling unit and the definition of households from which individual participants are sampled.

### Advantages of using satellite images for creating a sampling frame

In a constantly evolving world, sampling frames and household lists from previous censuses can rapidly become obsolete, requiring a closer-to-real-time sampling frame and household lists which better reflect the situation in which the study is conducted [62]. Our sampling frame creation method uses recent high resolution satellite images which are more representative of clusters and households in our study area. Obtaining the GPS coordinates of the selected households also makes it easier for data collectors to navigate to selected households and facilitates study conduct. It also circumvents bureaucratic delays in accessing census sampling frames, which allows for faster and better planning of research activities.

### Specific challenges in nomadic populations

The sampling methods that have historically been used to study nomadic populations are at risk of selection bias and could result in biased disease estimates [22, 25–28, 52]. This is particularly relevant for snakebite because the spatial location of the bite incident is necessary in evaluating the risk of snakebite and linking this risk to ecological factors such as altitude, snake species and habitats, and forest cover. Approaches such as the waterpoint approach may correlate with snake population density, especially in arid and semi-arid regions. There is emerging evidence that distance to water sources may predict the risk of snakebite in some communities [45].

Despite the multitude of definitions of a household in epidemiological research (sleeping under a roof, sharing a meal, minimum distance between houses, etc) [63], households in settled populations remain relatively constant and events such as births, deaths or travel can be identified and accounted for [8]. In mobile nomadic populations, households are fluid and some households split into two and one group takes some assets that include livestock and migrate in search of fertile grazing lands. These movements are harder to predict, track and account for and they can reduce the power of a fixed sample of households [8]. Approaches such as the camp approach, enumeration area and social structure approach are likely to capture only a fraction of the true mobile populations. For example, Weibel and collaborators collected digital fingerprints of 933 women in mobile pastoral camps in Chad, but despite revisiting the same location four times, they encountered only 22 (2.4%) women twice [31]. Furthermore, these movements can bias the effect of household level risk factors. For example, in split households, using assets to measure wealth can result in over-estimation of poverty and can bias the association with snakebite envenoming.

### Novelty and feasibility of our approach

A similar method was described for studying semi-nomadic populations in Naitolia, Tanzania [64]. The study was conducted on a less mobile agro-pastoralist population, used subscription imaging software, neither used sampling grids nor a multistage sampling strategy, and had a smaller sampling frame, containing a total of 307 households [64]. We combined contemporary geospatial and epidemiological techniques and applied them to a much larger population. Our sampling strategy can be conducted entirely digitally using open-source satellite imaging and GIS software. It provides recent information on clusters and households in the study area, and there are fewer bureaucratic delays or bottlenecks that would have been associated with gaining access or permission to census household lists. A drawback of this approach is the time and manual effort required to digitally identify and tag all households in the study area. The use of GPS coordinates to locate selected households during the data collection phase can partially offset some of the time spent in earlier stages.

### Limitations/lessons learned

Researchers interested in using our approach to conduct epidemiological surveys should consider the following limitations when designing their studies. Data collection was conducted three months after the geospatial mapping phase. In highly mobile populations, such delays could reduce the chances of identifying households in their originally mapped locations. We had a limited ability to ascertain that a structure that looked like a homestead, was indeed a household, and it was uncertain which ones were inhabited.

Data collection was conducted between the months of December and March, which falls within the dry season. Mobile pastoralists populations are known to move more frequently and to cover longer distances during the dry season, thereby reducing the chances of finding households in their originally mapped locations. Despite these limitations, we correctly identified 65.4% of households using our approach. We recommend shortening the delay between geospatial mapping and data collection if feasible. Mobile pastoralists move less frequently and cover shorter distances during the rainy seasons, but data collection in the rainy season is hampered by limited accessibility and risk of flash floods and other adverse weather events.

### Future perspectives

This paper addresses the need for adaptive and flexible epidemiological methods and techniques to study highly mobile populations. Contemporary global issues such as conflict and climate health have important consequences on the health of those affected and we suggest an approach to conduct epidemiological surveys and reliably assess their health needs. Our methodology also provides a unique opportunity to collect human and animal data among mobile pastoralists populations, thereby employing a One Health approach to estimate the true burden of snakebite envenoming in these populations. Whilst we recognise the value of GIS techniques in epidemiological studies, we are unclear about the need for informed consent to collect and use the GPS coordinates of households of nomadic pastoralists. We recommend further research and guidance on ethical considerations that offer agency to nomads on whether they would like to have this information collected. We also recommend further research to demonstrate the superiority of this methodology by comparing outcomes such as accuracy, efficiency, or cost between studies using different methods to assess the burden of snakebite in nomadic populations.

## Conclusion

Epidemiological studies involving nomadic populations need to factor in the fluidity in geographic localisation and household definition that are characteristic of mobile pastoralist populations. Highly mobile populations require specific considerations in selecting or creating sampling frames and sampling units for epidemiological research. Snakebite risk has a strong spatial component and using census-based sampling frames would be inappropriate in nomadic populations. We propose using open-source satellite imaging and geographic information systems to improve the conduct of epidemiological research in these populations.

## Supporting information

S1 Checklist PLOS inclusivity in global research checklist. (DOCX)

S1 Table PubMed search terms for scoping review on survey methods in nomadic populations. (DOCX)

S2 Table PubMed search terms for scoping review of recent community-based studies on snakebite. (DOCX)

S1 Fig PRISMA Flow chart of article selection process for scoping review on survey methods in nomadic populations. (DOCX)

S2 Fig PRISMA Flow chart of article selection process for scoping review of recent community-based studies on snakebite. (DOCX)
