# Supplementary material for: Conducting epidemiological studies on snakebite in nomadic populations: A methodological paper
Source: PLoS Negl Trop Dis. 2023 Dec 28;17(12):e0011792. doi: 10.1371/journal.pntd.0011792 (PMC10754435; doi:10.1371/journal.pntd.0011792)
Supplement: S1 Table — (DOCX) [file pntd.0011792.s002.docx]

**S1 Table - PubMed search terms for scoping review on survey methods in nomadic populations**

| Number | Search Terms |
| --- | --- |
| 1 | Nomad* [Title/Abstract] OR pastoralist* [Title/Abstract] OR “transients and migrants” [MeSH] |
| 2 | Survey* [MeSH] OR survey [Title/Abstract] OR cross-sectional stud*[MeSH] OR cross-sectional stud* [Title/Abstract] |
| 3 | “Africa, Western” [MeSH] OR benin* [Title/Abstract] OR burkina fas* [Title/Abstract] OR cape verd* [Title/Abstract] OR cabo verd* [Title/Abstract] OR ivory coast [Title/Abstract] OR cote d’ivoire* [Title/Abstract] OR gambia* [Title/Abstract] OR ghana* [Title/Abstract] OR (guinea* NOT pig*) OR bissau [Title/Abstract] OR liberia* [Title/Abstract] OR (mali NOT fowl) OR malian [Title/Abstract] OR mauritania* [Title/Abstract] OR nigeria* [Title/Abstract] OR senegal* [Title/Abstract] OR sierra leon* [Title/Abstract] OR togo* [Title/Abstract] OR “Africa, Central” [MeSH] OR angola [Title/Abstract] OR cameroon* [Title/Abstract] OR cameroun [Title/Abstract] OR chad [Title/Abstract] OR tchad [Title/Abstract] OR congo* [Title/Abstract] OR DRC [Title/Abstract] OR equatorial guinea* [Title/Abstract] OR gabon* [Title/Abstract] OR Sao Tome [Title/Abstract] OR Principe [Title/Abstract] OR “Africa, Eastern” [MeSH] OR Burundi* [Title/Abstract] OR Comoros [Title/Abstract] OR Djibouti* [Title/Abstract] OR Eritrea* [Title/Abstract] OR Ethiopia* [Title/Abstract] OR Kenya*[Title/Abstract] OR “Africa, Southern” [MeSH] OR Madagascar [Title/Abstract] OR Malawi [Title/Abstract] OR Mauritius [Title/Abstract] OR Mayotte [Title/Abstract] OR Mozambique [Title/Abstract] OR Reunion [Title/Abstract] OR Rwanda* [Title/Abstract] OR Seychelles [Title/Abstract] OR Somalia* [Title/Abstract] OR Sudan* [Title/Abstract] OR Tanzania* [Title/Abstract] OR Uganda* [Title/Abstract] OR Zambia [Title/Abstract] OR Zimbabwe [Title/Abstract] OR botswana* [Title/Abstract] OR lesotho* [Title/Abstract] OR malawi* [Title/Abstract] OR mozambiq* [Title/Abstract] OR namibia* [Title/Abstract] OR swaziland [Title/Abstract] OR zambia* [Title/Abstract] OR Zimbabwe [Title/Abstract] OR morroc* [Title/Abstract] OR Tunisia [Title/Abstract] OR Egypt [Title/Abstract] OR Libya [Title/Abstract] OR Algeria [Title/Abstract] OR “south sudan” [Title/Abstract] OR “Africa, Northern” [MeSH] |
| 4 | 1 AND 2 AND 3 |
