# Supplementary material for: Conducting epidemiological studies on snakebite in nomadic populations: A methodological paper
Source: PLoS Negl Trop Dis. 2023 Dec 28;17(12):e0011792. doi: 10.1371/journal.pntd.0011792 (PMC10754435; doi:10.1371/journal.pntd.0011792)
Supplement: S2 Table — (DOCX) [file pntd.0011792.s003.docx]

**S2 Table - PubMed search terms for scoping review of recent community-based studies on snakebite**

| Number | Search Terms |
| --- | --- |
| 1 | Snakebite* [Title/Abstract] OR “snake envenoming”* [Title/Abstract] OR "Snake Bites"[MeSH Terms] |
| 2 | Survey* [MeSH] OR survey [Title/Abstract] OR cross-sectional stud*[MeSH] OR cross-sectional stud* [Title/Abstract] |
| 3 | 1 AND 2 |
| 4 | Publications in the last 10 years – 2012 to 2022 |
