# Supplementary material for: Conducting epidemiological studies on snakebite in nomadic populations: A methodological paper
Source: PLoS Negl Trop Dis. 2023 Dec 28;17(12):e0011792. doi: 10.1371/journal.pntd.0011792 (PMC10754435; doi:10.1371/journal.pntd.0011792)
Supplement: S2 Fig — (DOCX) [file pntd.0011792.s005.docx]

**S2 Fig - PRISMA Flow chart of article selection process for scoping review of recent community-based studies on snakebite**

**Identification of studies via databases and registers**

Records removed *before screening*:

Duplicate records removed (n = 0 )

Records identified from:

PubMed (n = 184 )

**Identification**

Records excluded: (n = 160)

Wrong study design (n=117)

Wrong study population (43)

Records screened

(n = 184)

Reports sought for retrieval

(n = 24)

Reports not retrieved

(n = 0)

**Screening**

Reports excluded: (n = 6)

Studies not reporting community-level incidence or prevalence of snakebite (n = 5)

Studies conducted using the same dataset as a study included in review (n = 1)

Reports assessed for eligibility

(n = 24)

Studies included in review

(n = 18)

**Included**

**References**

1. Page MJ, McKenzie JE, Bossuyt PM, Boutron I, Hoffmann TC, Mulrow CD, et al. The PRISMA 2020 statement: an updated guideline for reporting systematic reviews. BMJ 2021;372:n71. doi: 10.1136/bmj.n71
